# A Novel Adhesion Molecule in the Murine Thymic Microenvironment: Functional and Biochemical Analysis

**DOI:** 10.1155/1992/18016

**Published:** 1992

**Authors:** Nesrina Imami, Heather M. Ladyman, Eugenia Spanopoulou, Mary A. Ritter

**Affiliations:** 1 Department of Immunology Royal Postgraduate Medical School Hammersmith Hospital Du Cane Road London W12 ONN UK; 2 National Institute for Medical Research Mill Hill London NW71AA UK

**Keywords:** Thymus, thymocytes, lymphocyte activation, PI linkage

## Abstract

The rat monoclonal antibody (mAb) 4F1, raised against mouse thymic stromal cells, recognizes cortical epithelium in tissue sections of mouse thymus; however, in flow cytometry, activated leucocytes (T cells, B cells, and macrophages) and transformed thymocytes are also positive for the 4F1-antigen (4F1-Ag). Western blotting, under both reducing and nonreducing conditions, demonstrates that the molecule to which 4F1 binds is expressed in four forms, 29, 32, 40, and 43 kD, all of which carry N-linked carbohydrate; and that the structure is identical on epithelium and lymphocytes. The 4F1-Ag on cortical epithelium is partially sensitive to PI-PLC treatment, whereas on transformed epithelial and lymphoid cell lines, it was resistant to this enzyme. The molecule, therefore, may exist in both transmembrane and phosphoinositol-linked forms. In functional blocking experiments, mAb 4F1 gave inhibition of both T-cell proliferation in MLR and of cytotoxic T-cell killing of alloantigenic targets; it also blocked adhesion of transformed thymocytes to thymic epithelial cells *in vitro*. These molecular and functional characteristics suggest that the 4F1-Ag is a novel adhesion molecule that may be involved both in intrathymic T lymphocyte differentiation and in peripheral T-cell function.


*Developmental Immunology*, 1992, Vol. 2, pp. 161–173
Reprints available directly from the publisher
Photocopying permitted by license only

© 1992 Harwood Academic Publishers GmbH
Printed in the United Kingdom

### A Novel Adhesion Molecule in the Murine Thymic Microenvironment: Functional and Biochemical Analysis

NESRINA IMAMI*, HEATHER M. LADYMAN, EUGENIA SPANOPOULOU†, and MARY A. RITTER

*Department of Immunology, Royal Postgraduate Medical School, Hammersmith Hospital, Du Cane Road, London W12 0NN; †National Institute for Medical Research, Mill Hill, London NW7 1AA*

The rat monoclonal antibody (mAb) 4F1, raised against mouse thymic stromal cells, recognizes cortical epithelium in tissue sections of mouse thymus; however, in flow cytometry, activated leucocytes (T cells, B cells, and macrophages) and transformed thymocytes are also positive for the 4F1-antigen (4F1-Ag). Western blotting, under both reducing and nonreducing conditions, demonstrates that the molecule to which 4F1 binds is expressed in four forms, 29, 32, 40, and 43 kD, all of which carry N-linked carbohydrate; and that the structure is identical on epithelium and lymphocytes. The 4F1-Ag on cortical epithelium is partially sensitive to PI-PLC treatment, whereas on transformed epithelial and lymphoid cell lines, it was resistant to this enzyme. The molecule, therefore, may exist in both transmembrane and phosphoinositol-linked forms. In functional blocking experiments, mAb 4F1 gave inhibition of both T-cell proliferation in MLR and of cytotoxic T-cell killing of alloantigenic targets; it also blocked adhesion of transformed thymocytes to thymic epithelial cells *in vitro*. These molecular and functional characteristics suggest that the 4F1-Ag is a novel adhesion molecule that may be involved both in intrathymic T lymphocyte differentiation and in peripheral T-cell function.

KEYWORDS: Thymus, thymocytes, lymphocyte activation, PI linkage.

### INTRODUCTION

It is now known that T lymphocyte differentiation takes place within the microenvironment of the thymus, although the precise inductive and selective mechanisms whereby stromal cells of epithelial and haemopoietic origin are involved remain yet to be resolved (Marrack and Kappler, 1987; von Boehmer, 1988).

Although specific signals that induce cellular differentiation in the thymus have not been identified, T-cell development requires close interaction between thymic microenvironmental cells (epithelium, dendritic cells, and macrophages) and the developing lymphocytes (Stutman et al., 1969). This interaction is likely to take two main forms: first, direct cell–cell contact involving cell-surface molecules, such as antigen receptors and MHC molecules, as well as accessory/adhesion molecules such as CD2, CD4, CD8, and LFA-3 (CD58); and second, interaction between soluble molecules, such as cytokines, and their cell-surface receptors (Bierer et al., 1989; Springer, 1990).

A recent approach to the analysis of these intrathymic mechanisms has been to raise monoclonal antibodies (mAb) to molecules in/on thymic stromal cells (DeMaagd et al., 1985; Kanariou et al., 1989). These reagents, raised to both human and rodent thymus, have revealed considerable heterogeneity within the epithelial component of the mammalian thymic microenvironment. One of these antibodies, 4F1, binds to all cortical epithelium and small patches of epithelial cells in the medulla of mouse thymus (Kanariou et al., 1989), and in the recent workshops on thymic epithelial antibodies has been defined as a CTES (cluster of thymic epithelial staining) III reagent (Kampinga et al., 1989; Ladyman et al., 1991).

In this paper, we describe structural and functional analysis of the molecule to which 4F1 binds (4F1-Ag). Our data indicate that the 4F1-Ag may represent a novel adhesion molecule that is

*Corresponding author.

involved in both intrathymic T lymphocyte differentiation and peripheral T-cell function.

### RESULTS

### Distribution and Expression of the 4F1-Ag

The 4F1 rat mAb has previously been shown to label strongly all cortical epithelium and to give weak staining of small patches of epithelium in the medulla of mouse thymus (Kanariou et al., 1989).

Immunocytochemical staining of the Thy-*myc* transgene-derived thymoma cell lines TM25.F1 (epithelial), TM25.103, and TM25.114 (mixed epithelial and lymphoid) (Spanopoulou et al., 1989) confirmed the reactivity of 4F1 with epithelial cells, but showed that transformed lymphoid cells were also strongly positive (Fig. 1). These cells provided a useful source of antigen. Flow cytometric analysis showed high expression of 4F1-Ag on the surface of TM25.114 lymphocytes (Table 1). The molecule is also expressed on the BW 5147 thymoma line (lymphoid cells only) and on epithelial thymoma cells grown in isolation from any lymphocytes (TM25.F1). Thus, both lymphoid and epithelial cells must be capable of synthesising the molecule *de novo*. Although most samples of normal thymocytes were found to be 4F1-negative, an occasional thymus (2/14) was strongly positive (Table 1; Fig. 3), suggesting that the 4F1-Ag may be up-regulated under certain conditions such as infection/activation and transformation.

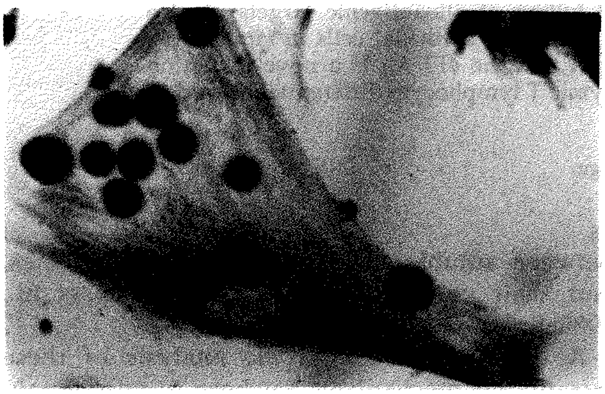

(a)

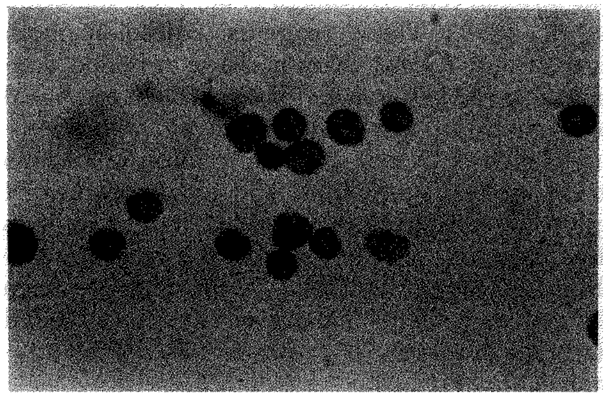

(b)

FIGURE 1. Immunoperoxidase staining of mouse thymoma cell line (TM25.114): (a) mAb 4F1: both epithelial cells and lymphocytes are positive; (b) anti-Thy-1: only lymphocytes are labeled.

### Kinetics of Expression of 4F1-Ag on Splenocytes in an MLR

The 4F1-Ag is present on 4–6% of resting splenocytes. However, during the first 48 hr of an MLR this rises to 20%, and by 70 hr peak expression is reached (35% of cells are 4F1+). After this, the percentage of cells expressing the 4F1-Ag decreases rapidly (Fig. 2).

For one experiment, two-color flow cytometric analysis of MLR cells was performed at 72 hr when 30% of cells were 4F1-positive, and at 96 hr when only 14% of cells were 4F1-positive. Double immunofluorescence staining shows that several different subpopulations of leucocytes express this molecule during an MLR. These include some T cells, some B cells, and the majority of MHC class-II bearing cells (B cells and macrophages) (Table 2; Fig. 3). Similar data were obtained in subsequent repeat experiments.

### Functional Analysis of the 4F1-Ag: Lymphocyte-Epithelial Cell Adhesion

Addition of 4F1 to mixed cultures of transformed

TABLE 1

Expression of the 4F1 Antigen[a]

| Cells | % cells stained | | | | |
|---|---|---|---|---|---|
| | IA | 4F1 | Thy-1.2 | CD4 | CD8 |
| TM25.114 | 2.7±2 | 70.3±9 | 75.5±8 | 76.7±16 | 69.9±10 |
| TM25.120 | 2.5±1 | 72.1±6 | 68.4±2 | 49.1±4 | 49.8±3 |
| TM25.103 | 1.5±1 | 74.9±7 | 93.0±1 | 56.3±12 | 51.7±5 |
| BALB/c NT[b] | 47.4 | 80.8 | 77.9 | 79.8 | 77.0 |

[a]Thymoma lymphoid cells and normal BALB/c thymocytes ($10^6$) were stained in suspension using the immunofluorescence technique and analyzed by flow cytofluorimetry. The percentage of cells stained by other mAbs such as anti-IA, anti-CD4, anti-CD8, and anti-Thy1.2 are shown in comparison. Data from three independent experiments (mean %±standard deviation).
[b]Typical of the 2/14 mice.

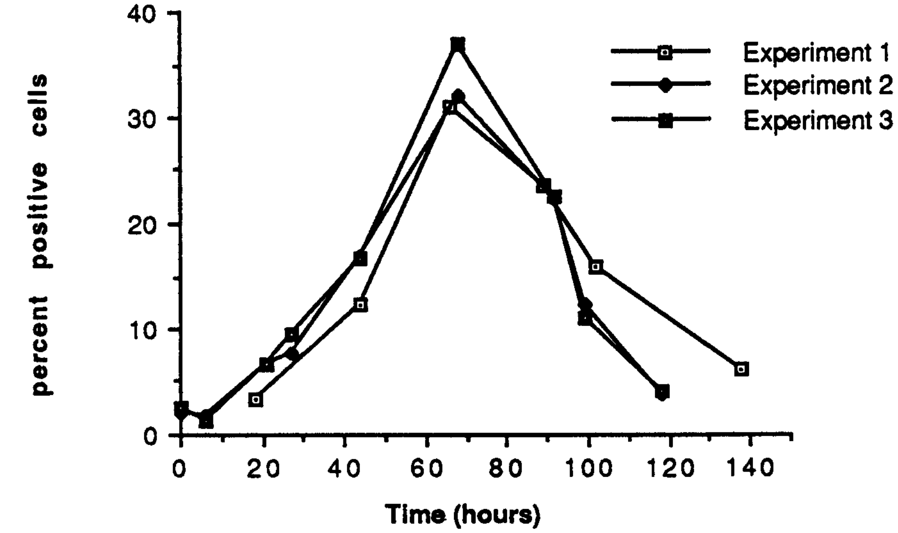

FIGURE 2. Kinetics of expression of the 4F1-Ag in a BALB/c v. CBA splenocyte MLR. At different time intervals, aliquots of cells were removed from the culture, labeled in suspension immunofluorescence with mAb 4F1 or a negative control mAb IVC4, and analyzed by flow cytometry. Data from three experiments are shown.

thymocytes and thymic epithelial monolayers inhibited the adhesion that normally occurs between the two cell types, whereas an isotype-matched control mAb (IVC4) had no effect (Fig. 4). This inhibition was observed at all time points analyzed (12, 24, and 48 hr). These data indicate that the 4F1-Ag may be important in the interaction between developing thymocytes and their epithelial microenvironment.

### Functional Analysis of the 4F1-Ag: Inhibition of MLR

In the second assay system, 4F1, but not the isotype-matched control mAb (IVC4), totally inhibited the proliferative response of mature peripheral T cells in an MLR in a dose-dependent manner (Fig. 5). The 4F1 mAb, therefore, appears to recognize a molecule that is important in T-cell adhesion and activation.

TABLE 2
Two-color Flow Cytometric Analysis of MLR Cells at 72 and 96 Hours[a]

| Cell subset | % of subset 4F1+ (72 hr) | % of subset 4F1+ (96 hr) |
|---|---|---|
| LCA (CD45) | 38 | 9 |
| MHC class II (IA) | 60 | 25 |
| Thy-1 | 26 | 13 |
| B cells | NT | 8 |

[a]Cells were labeled in suspension immunofluorescence with IgM antibody 4F1-FITC together with one of the IgG antibodies indicated. The IgG antibodies were detected using isotype-specific secondary reagents [PE-F(ab')$_2$ rabbit antirat IgG].

### Functional Analysis of the 4F1-Ag: Inhibition of Cytotoxic Killing

Observations that the mAb blocked the alloreactive response and that the molecule was up-regulated during an MLR led to experiments in which the effect of 4F1 on the cytotoxic killing was studied. When 4F1 was added in cultures during the $^{51}$Cr release assay, it was found to block completely the killing of radiolabeled target cells by alloreactive cytotoxic T lymphocytes, whereas the isotype-matched control mAb (IVC4) had no effect (Fig. 6).

### Biochemical Analysis of 4F1-Ag

The following cell types were analyzed by Western blotting and immunoprecipitation: epithelial thymoma cells (line TM25.F1), normal thymic epithelium, lymphoid thymoma lines TM25.114 and BW5147, and normal thymocytes. The 4F1-Ag was found to have the following properties (Fig. 7):

1. There are up to four bands that run together in two pairs.

2. The molecular weights of the upper two bands are approximately 43 and 40 kD, and those of the lower two bands are approximately 32 and 29 kD.

3. These bands have the same characteristics under both reducing and nonreducing conditions.

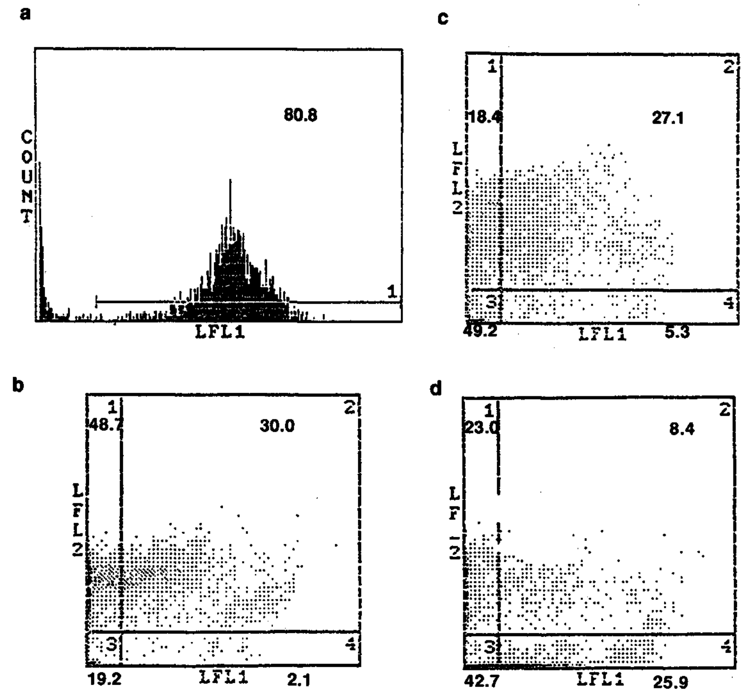

FIGURE 3. Flow cytometric analysis: (a) Single staining of normal BALB/c thymocytes. (b–d) Two-color flow cytometry: (b) 4F1+anti-CD45; (c) 4F1+anti-MHC class II; and (d) 4F1+anti-Thy-1. X axis: 4F1-FITC staining; Y axis: second antibody phycoerythrin staining.

4. The same bands are seen for both lymphoid and epithelial cells.

5. The expression of individual bands is variable, but this shows no correlation with cell type (see Figs. 7, 8, and 9), and is seen in both the presence and absence of protease inhibitors.

6. A ladder pattern is observed behind the bands throughout the gel, and for all cell types analyzed. However, we have also observed this with other IgM mAbs.

Glycosylation of the 4F1-Ag was analyzed using either tunicamycin in culture or by treating cells with endoglycosidase F (endo F). When cells were grown in the presence of tunicamycin, there was a reduction in the intensity of the two upper bands (Fig. 8). However, in our system, the presence of tunicamycin, even at 2 $\mu$g/ml, blocked adherence of the cells to each other and to the tissue-culture flasks and appeared to inhibit their growth. Further studies of glycosylation, therefore, were performed using endo F. After treatment of cells with this enzyme, only the 43-kD band remained, and this was considerably reduced in intensity (Fig. 9). Treatment with O-Glycanase had no effect on any of the bands.

Analysis of membrane insertion of the 4F1-Ag on TM25.F1 epithelial cells using PI-PLC showed that there was no difference between the Western blots of treated and untreated cells. Similarly, PI-PLC had no effect on transformed lymphocytes (TM25.114 and BW5147) when treated in suspension prior to analysis by flow cytometry. In contrast, PI-PLC treatment of fresh frozen thymic tissue sections showed a partial but significant reduction in the fluorescence intensity of 4F1 staining (Table 3; Fig. 10). The effect of PI-PLC on Thy-1 staining (positive control, known to be PI-linked) was considerably greater, whereas the enzyme had no effect on either MHC class–II or IVC4-labeling intensities (negative controls, not PI-linked).

### DISCUSSION

The aim of the work presented in this paper was to explore the nature and function of the molecule detected by mAb 4F1. This mAb was initially raised against cortical epithelium of mouse thymus. However, our data indicate that the 4F1-Ag is present both within the thymus and in the periphery, and that it may be involved in T-cell activation as well as in cell–cell interactions between lymphoid cells and their stromal-cell microenvironment.

Immunohistochemical and flow cytometric analyses have revealed that the 4F1-Ag is present at high levels on normal thymic cortical epithelium, transformed epithelial and lymphoid-cell lines, and on activated T and B lymphocytes. Expression on other leucocytes (macrophages/dendritic cells) is up-regulated by T-cell activation. Addition of the 4F1 mAb to mixed thymoma cell cultures blocked the adhesion of transformed CD4+,CD8+ "double positive" cortical-type thymocytes to 4F1++, IVC4+/− cortical-type epithelium (Kanariou et al., 1989; Spanopoulou et al., 1989), suggesting a role for the 4F1-Ag in the interaction of developing thymocytes with their microenvironment. The mAb also blocked T-cell proliferation in an MLR and cytotoxic T-cell killing of alloantigenic targets, indicating a further role for the 4F1-Ag in activation/interaction of peripheral T lymphocytes with their targets.

The 4F1-Ag is a glycoprotein expressed in four forms; with apparent molecular weights of approximately 29, 32, 40, and 43 kD, and is identical in lysates from both epithelium and lymphocytes. Because the same pattern is obtained under reducing and nonreducing conditions, the four

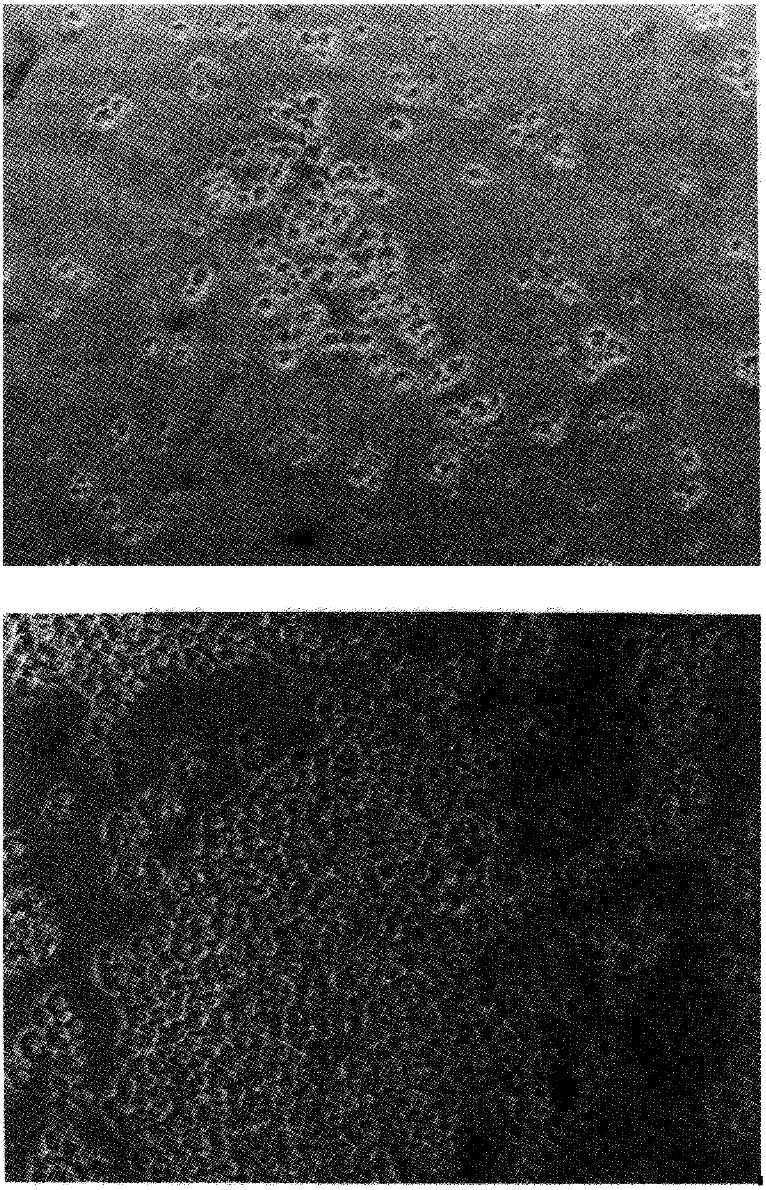

(a)

(b)

FIGURE 4. (a) Addition of 4F1 to mixed cultures of transformed thymocytes and thymic epithelial monolayers (TM25.114) inhibited the adhesion that normally occurs between the two cell types. (b) An isotype-matched control mAb (IVC4) had no effect.

chains identified are not associated covalently with each other. In addition, because all four bands are detected by Western blotting, each polypeptide must carry the 4F1 epitope. The multiple bands are therefore likely to represent isoforms of the same molecule. These could result from differential transcription off a single gene, leading to differences in either the external portion of the molecule, as in CD45, or in its method of membrane insertion, as in LFA-3 (Streuli et al., 1987; Springer, 1990). Alternatively, the presence of several bands could reflect differences in glycosylation, as has been observed for Thy-1 (Williams, 1988). It is possible that some may represent immature internal forms.

The partial sensitivity of the 4F1-Ag on normal epithelial cells to cleavage by the enzyme PI-PLC suggests that some 4F1 molecules are PI-linked while others may not be. Unfortunately, our attempts to analyze the membrane linkage of individual isoforms using transformed epithelial and lymphoid cell lines were unsuccessful, with all four polypeptides showing resistance to PI-PLC cleavage. A similar resistance has been described for the PI-linked Thy-1 and Ly-6 molecules after activation or transformation of murine T cells (Low et al., 1988; Presky et al., 1990); modification of the PI linkage by palmitoylation has been proposed to be the underlying mechanism responsible. We are, therefore, currently establishing an alternative system for analyzing the biochemistry of cell-surface 4F1-Ag isoform attachment on normal epithelium using multiple sections of fresh frozen thymus and primary epithelial monolayer cultures. The existence of two membrane anchorage forms for a single molecule has also been observed for LFA-3 (CD58), NCAM (CD56), and Leu 8 (p90 MEL-14),

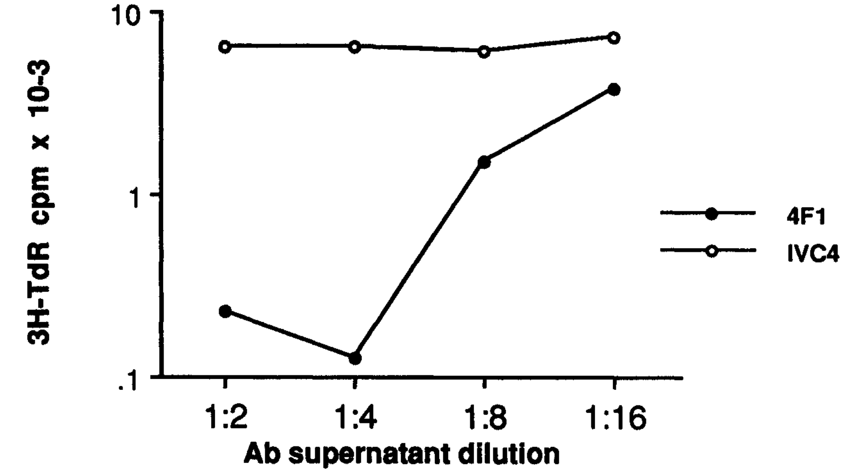

FIGURE 5. 4F1 mAb inhibits the proliferative response of H-2$^d$ splencoytes in a MLR (H-2$^d$ v. H-2$^{bxk}$) in a dose-dependent fashion. The isotype-matched control mAb (IVC4) had no effect.

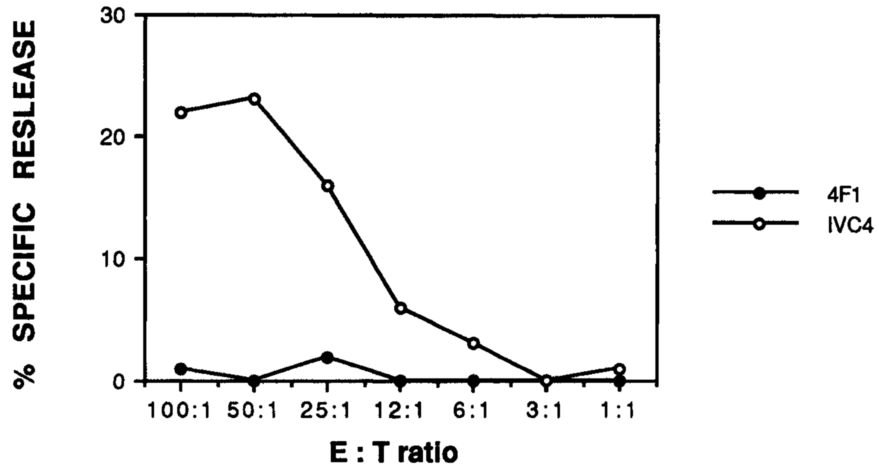

FIGURE 6. 4F1 mAb inhibits the cytotoxic killing of allogeneic targets (H-2$^d$ v. H-2$^k$) during the $^{51}$Cr release assay. The isotype-matched control has no inhibitory effect.

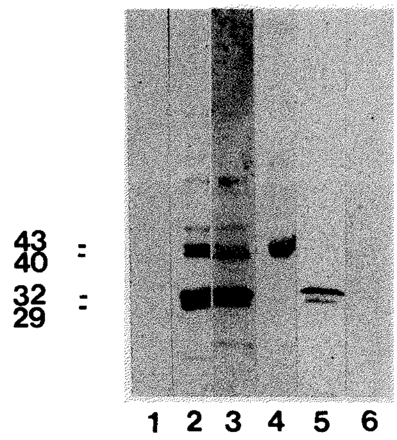

FIGURE 7. Western blotting of the 4F1-Ag. Cell lysates were subjected to SDS-10%PAGE and immunoblotting. TM25.F1 cells: isotype-matched control mAb (IVC4, lane 1), 4F1 (lanes 2 and 3). Mouse thymic stroma: 4F1 (lanes 4 and 5; two different lysates), IVC4 (lane 6).

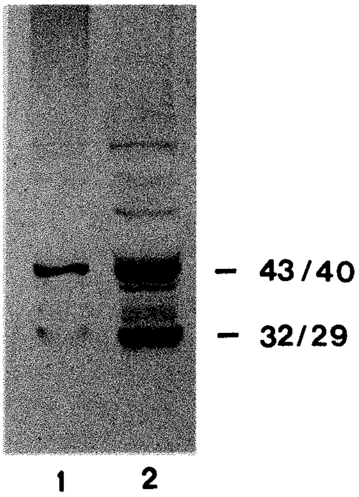

FIGURE 9. Endoglycosidase F treatment of BW5147 cells. BW5147 cells were treated with either endo F (lane 1) or control buffer (lane 2). Cell lysates were prepared and subjected to SDS-10%PAGE and immunoblotting.

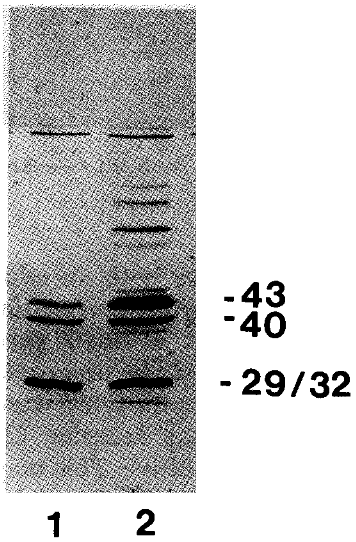

FIGURE 8. Tunicamycin treatment of TM25.F1 cells. TM25.F1 cells were grown in the presence (lane 1) or absence (lane 2) of tunicamycin. Cell lysates were prepared and subjected to SDS-10%PAGE and immunoblotting.

TABLE 3
PI-PLC Treatment of Mouse Thymus Sections[a]

(a) Data from 4F1 PI-PLC treatment statistics[b]
Paired samples

| Variable | −PI-PLC | +PI-PLC |
|---|---|---|
| Mean | 1.298 | 1.494 |
| Standard deviation | 0.168 | 0.168 |
| Paired observations | 8 | 8 |

(b) Data from Thy-1 PI-PLC treatment statistics[c]
Paired samples

| Variable | −PI-PLC | +PI-PLC |
|---|---|---|
| Mean | 2.486 | 3.341 |
| Standard deviation | 0.144 | 0.689 |
| Paired observations | 8 | 8 |

[a]Frozen unfixed sections were treated with or without PI-PLC enzyme, washed, fixed, and processed for immunofluorescence using 4F1, anti-Thy1.2 (as positive control) and anti-IA (as negative control). Fluorescence intensity was measured using an automatic exposure meter attached to the Olympus BH2 fluorescence microscope. PI-PLC treatment causes a significant reduction in fluorescence intensity.

[b]t-statistics: −2.591; degrees of freedom: 7; significance 0.036. The difference is statistically significant ($p<0.05$).

[c]t-statistics: −3.630; degrees of freedom: 7; significance: 0.008. The difference is statistically significant ($p<0.01$).

although the significance of a PI-versus-transmembrane linkage is currently unclear (Presky et al., 1990; Springer, 1990).

Analysis of the glycosylation of the 4F1 molecule showed that both tunicamycin and endo F treatment resulted in a reduction in the intensity of some 4F1-Ag bands. However, no lower molecular weight band representing a deglycosylated form appeared, nor was the smallest form (29 kD) enhanced. The most likely explanation of these data is that the 4F1 epitope is dependent upon the presence of carbohydrate. This is supported by the fact that the isotype of the 4F1 mAb is IgM, characteristic of an anticarbohydrate response. It is also consistent with observations that glycosylation seems to be a characteristic of molecules that are important for the execution of the functions such as adhesion and transmembrane signaling (Krensky et al., 1983; Dustin et

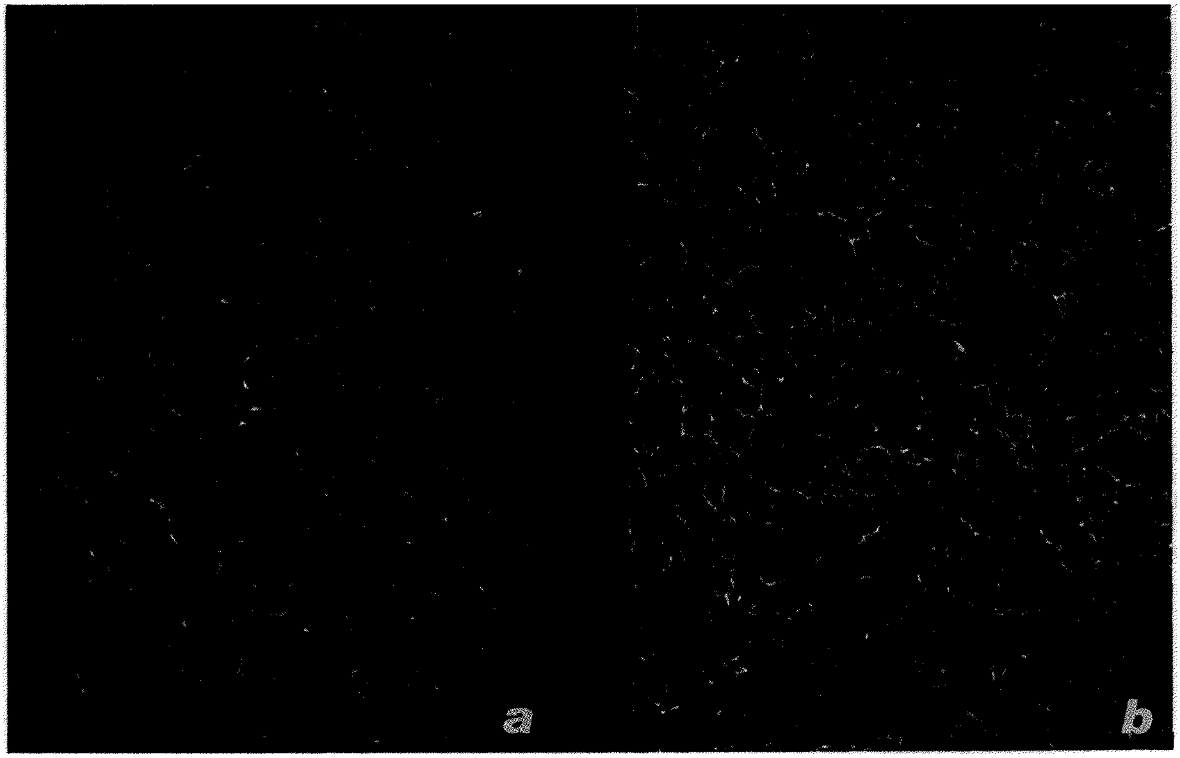

FIGURE 10. Immunofluorescence staining of frozen sections of normal mouse thymus with mAb 4F1 visualized with FITC-conjugated rabbit antirat Ig after treatment with PI-PLC (a); and control buffer (b).

al., 1987; Seed, 1987). Interestingly, endo F only partially affected the 43-kD band, although this was strongly reduced by tunicamycin. Endo F is known to catalyze the hydrolysis of the glycosidic bonds of the chitobiose core structure of many high-mannose and biantennary complex Asn-linked oligosaccharides. However, hybrid structures containing bisecting (peripheral) GlcNAc-linked beta-(1,4) to the mannose core, and tri- and tetraantennary complex chains are resistant to endo F. The 43-kD isoform may therefore carry some complex carbohydrate of this type. In contrast, treatment with O-Glycanase had no effect on any band. O-Glycanase enzyme catalyzes the release of the Gal-beta-(1,3)GalNAc core disaccharide attached to serine or threonine residues of glycoproteins to give free oligosaccharides and an unsubstituted serine or threonine group. This type of carbohydrate appears to be absent from the 4F1-Ag.

The fact that the molecule is present on isolated cell lines of lymphoid and epithelial thymoma origin shows that the molecule is not acquired passively by either cell type, but is produced independently by both. In addition to its presence on cortical thymic epithelium, the molecule also appears on a small percentage of resting splenocytes and is up-regulated upon activation. Although thymocytes were normally 4F1-negative, occasional samples (2/14) were found to be strongly positive. This may reflect an acute infection in these animals, with involution of the thymic cortex and high expression on mature medullary lymphocytes.

Our data indicate that the 4F1-Ag is a novel molecule, because its molecular weight is not matched by any of those listed in the current "CD classification of leukocyte antigens" (Knapp et al., 1990). Although there are several well-described adhesion molecules such as LFA-3 (CD58), ICAM-1 (CD54), Leu 8 (p90 MEL-14), Pgp-1 (CD44), and NCAM (CD56) (Sadoul et al., 1986; Marlin and Springer, 1987; Springer et al., 1987; Sanders et al., 1988; Camerini et al., 1989), only LFA-3 shares similar distribution, structure, and functional characterstics with the 4F1 molecule, because it is up-regulated upon activation (Krensky et al., 1983; Sanders et al., 1988), has a similar range of relative molecular weight (Dustin et al., 1987), and is anchored into the cell membrane via both the PI-linked and transmembrane forms (Dustin et al., 1987; Seed, 1987). However, 4F1-Ag differs from LFA-3 in that it is expressed on cortical thymic epithelium, but not in the medulla, and LFA-3 is present on both cortical and medullary epithelium (with stronger expression in the medulla) (Singer and Haynes, 1987; our unpublished observations). 4F1-Ag is absent from red blood cells and endothelium (personal observations) contrary to LFA-3 (Dustin et al., 1987). Biochemical analysis of 4F1-Ag gives four discrete bands (29, 32, 40, and 43 kD) and that of LFA-3 gives a broad band around 65 kD (surface) and 35/39 kD and 37/41 kD (internal) (Dustin et al., 1987). Unfortunately, the 4F1 mAb does not perform well in immunoprecipitation, making it difficult to study internal versus surface forms; this may reflect a low affinity for solubilized antigen, characteristic of IgM antibodies.

Functional blocking studies with antibodies to human LFA-3 and murine 4F1-Ag show that both reagents block T-cell proliferation in an MLR and T-cell killing of alloantigenic target cells. On the other hand, although antibodies to LFA-3 block both the binding of fresh thymocytes to primary thymic epithelial cell cultures and the epithelial cell-induced proliferation of medullary thymocytes (CD1−,CD3+), our data show that 4F1 has no effect in this system although it does block binding of cortical-type thymocyte and epithelial cell lines (Singer and Haynes, 1987). Thus, on balance, the data indicate that the 4F1-Ag is a novel adhesion molecule and not the murine homologue of human LFA-3; however, it is possible that some biochemical and cell distribution differences could reflect species variation rather than a difference of identity. Future studies will focus on cloning the gene that encodes the 4F1-Ag and identification of its human homologue.

### MATERIALS AND METHODS

### Source of Tissue and Cell Lines

*Fresh thymocytes*

Thymus tissue obtained from young adult BALB/c, C57Bl/6, and CBA mice was thoroughly teased. Small fragments were allowed to sediment and supernatant thymocytes were washed in HEPES (20 mM) buffered RPMI 1640 (Flow, Scotland) before analysis.

*Fresh stromal cells*

The remaining stroma (sedimenting fragments) were further processed by digestion in 1.0 mg/ml collagenase (Sigma, UK) in RPMI with 20% FCS for 4 hr. Cells were then washed three times and layered over neat FCS for 1 hr. The interface was collected.

*Thymoma cell lines*

Epithelial cell line (TM25.F1) and mixed epithelial and lymphoid cell lines (TM25.114, TM25.103, TM25.120) derived from Thy-*myc* transgenic mice (generous gift of D. Kioussis; Spanopoulou et al., 1989) were used. The BW5147 thymoma lymphoid line was analyzed (ATCC, USA). All cells were cultured in RPMI 1640 supplemented with 10% FCS, L-glutamine (2 mM), sodium bicarbonate (2 g/l), penicillin (100 IU/ml) and streptomycin (100 $\mu$g/ml) (Flow).

### Source of Antibodies

4F1: MAb 4F1 is a rat IgM reagent that was raised to epithelial cells of the mouse thymic cortex (Kanariou et al., 1989). IVC4: mAb IVC4 is a rat IgM reagent that detects epithelial cells of the mouse thymic medulla (Kanariou et al., 1989). The antibodies were produced as tissue-culture supernatant. Additional monoclonal supernatant reagents were M5 114: rat antimouse Ia (MHC class II), IgG2b (Boehringer Mannheim; Bhattacharya et al., 1981); 30H-12: rat antimouse Thy-1.2, IgG2b (gift of R. Morris; Ledbetter and Herzenberg, 1979); YTS 191.1.2: rat antimouse L3/T4 (CD4), IgG2b (Sera-lab, UK); YTS 169.4: rat antimouse Lyt 2 (CD8), IgG2b (Sera-lab); M1/70.15: rat antimouse macrophage, IgG2b (Sera-lab); rat antimouse leukocyte common antigen (CD45; gift of J. Austyn); rat antimouse IgG (Serotec, UK). The following second-layer reagents were used: rabbit antirat Ig (Dakopatts, Denmark), FITC-rabbit antirat Ig (Dakopatts), HRP-rabbit antirat Ig (Dakopatts), phycoerythrin (PE)-affinity purified

$F(ab')_2$ rabbit antirat IgG (Serotec; cross reaction with mouse Ig removed).

### Immunoperoxidase Staining of Thymoma Lines

Thymoma cell lines were grown in slide flasks (Nunc) until confluent, washed extensively in serum-free Hepes-buffered RPMI at room temperature and then in phosphate buffered saline (PBS) for the last wash, fixed in methanol (1 min), and rinsed in PBS. Slides were blocked in 10% newborn calf serum (NCS) in PBS, incubated with primary antibody for 1 hr at 4 °C, followed by HRP-rabbit antirat Ig at 1:100 in PBS with 10% NCS, and then developed with diaminobenzidine (DAB) (6 mg/10 ml PBS with 5 $\mu$l of 30% $H_2O_2$/10 ml DAB solution). Slides were mounted in Kaiser's mountant.

### Flow Cytometry of Thymoma and MLR Cells

#### Single staining

Cells ($10^6$) were analyzed by suspension immunofluorescence for the expression of cell-surface molecules (Larche et al., 1988). After incubation with 100 $\mu$l of primary antibody for 1 hr at 4 °C, cells were washed twice in PBS, and incubated with the FITC-rabbit antirat Ig, diluted 1/10, for an additional 1 hr at 4 °C. Finally, cells were washed as before, and either analyzed immediately or fixed in ice cold 1% paraformaldehyde (Sigma) in PBS and analyzed within 24 hr by flow cytofluorimetry (EPICS profile, Coulter, USA).

#### *Double staining*

$10^6$ cells were incubated with 100 $\mu$l of 4F1-FITC or the isotype-matched control mAb for 30 min at 4 °C as described before, followed by incubation with a second antibody directed to CD45, IgG, Thy-1.2, CD8, CD4, MHC class II or macrophages. Finally, PE-affinity purified $F(ab')_2$ rabbit antirat IgG (Serotec) was added for a final 30-min incubation. Cells were washed in Hanks' buffer (Flow) without the phenol red and analyzed immediately by flow cytometry.

### Mixed Lymphocyte Reaction (MLR) and Proliferation Assay

Splenocytes from mice were teased into suspension, centrifuged over Lympho-Sep (Sera-lab), and the interface cells resuspended at $10^6$/ml. MLRs were performed using $10^5$ splenocytes of strain BALB/c (H-$2^d$) as responder cells stimulated with $0.5\times10^4$ irradiated (3000 rad) stimulator cells of strain T4 mice (H-$2^{kxb}$) in 0.2 ml per well in 96-well U-shaped plates (Flow) in the presence of different supernatant dilutions of either 4F1 or isotype-matched IVC4 control mAb. After 5 days in culture, each well was pulsed with 1 $\mu$Ci of tritiated methyl thymidine ([$^3$H]TdR; Amersham International, UK) for 8–16 hr and then harvested onto glass fiber filters. Proliferation as correlated with [$^3$H]TdR incorporation was measured by liquid scintillation spectroscopy. Results are expressed as the mean counts per minute (cpm) for triplicate cultures with percentage error of the mean <15%.

### $^{51}$Chromium Release/Cytotoxicity Assay

Cells from a one-way MLR were used as effector cells. Splenocytes were isolated, as described previously, from BALB/c and CBA mice and were cultured in 24-well plates at $10^6$ BALB/c v. $5\times10^5$ irradiated (3000 rad) CBA cells for 5 days.

#### *Generation and $^{51}$Cr labeling of target Con A blasts*

On days 2–3 of the MLR, splenocytes from a CBA mouse were isolated and 1 ml cultured in 24-well flat-bottomed tissue-culture plates at $1–2\times10^6$ cells/ml. Concavalin A (Con A; Pharmacia) at 2 $\mu$ g/ml was added per well, and gently resuspended. Cells were incubated at 37 °C for 72 hr and then washed three times in supplemented RPMI, counted and 10 $\mu$l of AB serum plus $^{51}$Cr at 200 $\mu$Ci/$10^7$ cells added. Cells were gently resuspended and incubated for 1 hr with occasional agitation. Meanwhile, the effector (MLR) cells were collected, washed twice, and resuspended at $10^7$/ml. Doubling dilutions were plated in 96-well round-bottomed tissue-culture plates (Flow) to give different effector-to-target (E:T) ratios starting from (100:1; $10^6$ effectors:$10^4$ target cells) downward. Target cells labeled with $^{51}$Cr were washed twice, counted, resuspended in complete medium, and added at a constant concentration of $10^4$/well.

Each dilution was done in triplicate. Two isotype controls (spontaneous, S; and total, T, $^{51}$Cr release), as well as antibody controls with effector and target cells from 100:1 downward, both in the presence and absence of the isotype-matched control mAb (IVC4), were included.

Spontaneous release (0% death) was assessed by adding 100$\mu$l of target cells to 100$\mu$l medium. Total release (100% death) was assessed by adding 100$\mu$l of target cells to 100$\mu$l of 1% Triton X-100 (Sigma)).

Plates were incubated for 4 hr and then centrifuged for 5 min at 1000 rpm. 100 $\mu$l of supernatant were removed from each well and placed in LP2 tubes (Luckhams). Each tube was sealed with wax and released $^{51}$Cr measured in a gamma counter. The mean of triplicate counts per minute (cpm) for each effector cell dilution was taken. Results are expressed as % specific release (%SR). This was calculated for each unknown (X) as follows:

$$\%SR = \frac{X\,(cpm) - S\,(cpm)}{T\,(cpm) - S\,(cpm)},$$

Data were plotted as the E:T ratio against the percentage specific release for that particular ratio.

### Two-way MLR—Kinetics of 4F1-Ag Expression

Spleen cells from BALB/c (H-2$^d$) and CBA (H-2$^k$) were teased into suspension, centrifuged over Lympho-Sep (Sera-lab), and the interface cells resuspended to 2×10$^6$/ml. 1 ml each of the BALB/c and CBA splenocyte suspensions was plated out into each 2-ml well of 24-well plates (Nunclon). Cells were sampled in triplicate at sequential time points from 0 to 142 hr and analyzed by suspension immunofluorescence and flow cytometry for expression of 4F1-Ag. Isotype-matched mAb IVC4 was used as a negative control.

### Western Blotting Analysis of 4F1-Ag

Cell lysates (10$^8$) or 1 cm$^3$ of tissue was prepared [10 mM TRIS/HCl, pH 7.4; 150 mM NaCl; 0.5% Nonidet P-40 (NP-40)] in the presence of protease inhibitor phenylmethylsulfonyl fluoride (1 mM PMSF; Sigma). Supernatant proteins were resolved (both under reducing and nonreducing conditions) by SDS-10% PAGE according to the modified discontinuous buffer system of Laemmli (1970) using a "mini-gel" apparatus (Hoefer Scientific Instruments). A mixture of standard protein markers (SDS-7B; Sigma) was used for the determination of relative molecular mass. Proteins were transferred from gels to nylon membrane using a wet electroblotter (Bio-Rad; Towbin et al., 1979), and membranes were stained for both 4F1 and the control mAb (IVC4) by indirect immunoperoxidase staining using HRP-rabbit antirat Ig (Dakopatts), and DAB substrate.

### Inhibition of Glycosylation of 4F1-Ag by Tunicamycin

TM25.F1 cells were grown in the presence of different concentrations (2–5 $\mu$g/ml) of tunicamycin (Sigma) in RPMI medium with 10% FCS. After 24 hr, whole cell lysates were prepared and subjected to Western blotting, as described before. Control cells were treated in an identical manner, omitting the tunicamycin.

### Enzyme Digestion of 4F1-Ag with Endoglycosidase F

Aliquots of cells (10$^8$ in 100 $\mu$l) were diluted to 600 $\mu$l with 0.1 M citrate-phosphate buffer, pH 6.0, and 100 mU of endo F (Sigma) were added for 6 hr at 37 °C (Newman et al., 1981). After digestion, cells were lysed and subjected to Western blotting, as described before. Control cells were treated in an identical manner, omitting the enzyme.

### Phospholipase C Treatment of TM25.F1 Cells

Phosphatidylinositol-specific phospholipase C (PI-PLC) isolated from *Bacillus thuringiensis*, with activity 336 $\mu$mol/min/ml, was used to treat whole TM25.F1 cells (Pierres et al., 1987). Cells (10$^8$) were incubated both in the presence or absence of PI-PLC (150 mU, Peninsula Laboratorie Europe) in PBS, pH 7.5, for 1 hr at 37 °C. Samples were washed thoroughly by centrifugation at high speed, and subjected to SDS-10% PAGE and Western blotting, as described before. One unit of enzyme activity is defined as the amount of enzyme that catalyzes the hydrolysis of 1 $\mu$mol of phosphatidylinositol per minute at pH 7.5 at 37 °C.

### PI-PLC Treatment of Mouse Thymus Sections and Thymoma Lymphoid Cell Lines in Suspension

Frozen unfixed sections were treated with PI-PLC enzyme (15 mU per section for 60 min at room temperature), washed, fixed, and processed for immunofluorescence using 4F1, anti-Thy1.2 (as positive control), and anti-Ia and IVC4 (as negative controls). Fluorescence intensity was measured using an automatic exposure meter attached to the Olympus BH2 fluorescence microscope (Olympus). Thymoma TM25.114 and BW5147 lymphoid cells were treated with or without PI-PLC (15 mU per $10^6$ cells) prior to suspension immunofluorescence staining and then analyzed by flow cytometry as described before.

*(Received July 31, 1991)*

*(Accepted September 23, 1991)*

### REFERENCES

Bhattacharya A., Dorf M.E., and Springer T.A. (1981). A shared alloantigenic determinant on Ia antigens encoded by the I-A and I-E subregions: Evidence for I region gene duplication. J. Immunol. **127:** 2488–2495.

Bierer B.E., Sleckman B.P., Ratnofsky S.E., and Burakoff S.J. (1989). The biological role of CD2, CD4 and CD8 in T-cell activation. Ann. Rev. Immunol. **7:** 579–599.

Camerini D., James S.P., Stamenkovic J., and Seed B. (1989). Leu-8/TQ1 is the human equivalent of the Mel-14 lymph node homing receptor. Nature **342:** 78–82.

DeMaagd R.A., Mackenzie W.A., Schuurman H.-J., Ritter M.A., Price K.M., Broekhuizen R., and Kater L. (1985). The human thymus microenvironment; heterogeneity detected by monoclonal anti-epithelial cell antibodies. Immunology **54:** 745–754.

Dustin M.L., Selvaraj P., Mattaliano R.J., and Springer T.A. (1987). Anchoring mechanisms for LFA-3 cell adhesion glycoprotein at membrane surface. Nature **329:** 846–848.

Kampinga J., Berges S., Boyd R., Brekelmans P., Colic M., van Ewijk W., Kendall M.D., Ladyman H., Nieuwenhuis P., Ritter M.A., Schuurman H.-J., and Tournefier A. (1989). Thymic epithelial antibodies: Immunohistochemical analysis and introduction of nomenclature. Summary of the Epithelium Workshop held at the 2nd Workshop "The Thymus." Histophysiology and Dynamics in the Immune System. Thymus **13:** 165–173.

Kanariou M., Huby R., Ladyman H., Colic M., Sivolapenko G., Lampert I., and Ritter M. (1989). Immunosuppression with Cyclosporin A alters the thymic microenvironment. Clin. Exp. Immunol. **78:** 263–270.

Knapp W., Dorken B., Gilks W.R., Rieber E.P., Schmidt R.E., Stein H., and von dem Borne A.E.G. Eds. (1990). Leucocyte typing IV. White cell differentiation antigens (Oxford: Oxford University Press).

Krensky A.M., Sanchez-Madrid F., Robbins E., Nagy J.A., Springer T.A., and Burakoff S.J. (1983). The functional significance, distribution, and structure of LFA-1, LFA-2, and LFA-3, cell surface antigens associated with CTL-target interactions. J. Immunol. **131:** 611–616.

Ladyman H., Boyd R., Brekelmans P., Colic M., van Ewijk W., von Gaudecker B., Kampinga J., Kendall M., Nieuwenhuis P., Schuurman H.-J., Tournefier A., and Ritter M. (1991). Monoclonal antiepithelial antibody workshop II flow cytometric analysis and biochemical analysis of thymic epithelial cell heterogeneity. *In:* Proceedings of the 10th international conference in immune reactions (New York: Marcel Dekker), in press.

Laemmli U.K. (1970). Cleavage of structural proteins during the assembly of the head of bacteriophage T4. Nature **227:** 680–685.

Larche M., Lamb J.R., O'Hehir R.E., Imami N., Zanders E.D., Quint D.E., Moqbel R., and Ritter M.A. (1988). Functional evidence for a monoclonal antibody that binds to the human IL-4 receptor. Immunology **65:** 617–622.

Ledbetter J.A., and Herzenberg L.A. (1979). Xenogenic monoclonal antibodies to mouse lymphoid differentiation antigens. Immunol. Rev. **47:** 63–90.

Low M.G., Stiernberg J., Waneck G.L., Flavell R.A., and Kincade P.W. (1988). Cell-specific heterogeneity in sensitivity of phosphatidylinositol-anchored membrane antigens to release by phospholipase C. J. Immunol. Methods **113:** 101–111.

Marlin S.D., and Springer T.A. (1987). Purified intercellular adhesion molecule-1 (ICAM-1) is a ligand for lymphocyte function-associated antigen 1 (LFA-1). Cell **51:** 813–819.

Marrack P., and Kappler J. (1987). The T cell receptor. Science **238:** 1073–1079.

Newman R.A., Sutherland R., and Greaves M.F. (1981). The biochemical characterisation of a cell surface antigen associated with lymphoblastic leukemia and lymphocyte precursors. J. Immunol. **126:** 2024–2030.

Pierres M., Naquet P., Barbet J., Marchetto S., Marics I., Devaux C., Barad M., Hyman R., and Rougon G. (1987). Evidence that murine hematopoietic cell subset marker J11d is attached to a glycosyl-phosphatidylinositol membrane anchor. Eur. J. Immunol. **17:** 1781–1785.

Presky D.H., Low M.G., and Shevach E.M. (1990). Role of phosphatidylinositol-anchored proteins in T cell activation. J. Immunol. **144:** 860–868.

Sadoul K., Meyer A., Low M.G., and Schachner M. (1986). Release of the 120 kDa component of the mouse neural cell adhesion molecule N-CAM from cell surfaces by phosphatidylinositol-specific phospholipase C. Neurosci. Lett. **72:** 341–346.

Sanders M.E., Makgoba M.W., Sharrow S.O., Stephany D., Springer T.A., Young H.A., and Shaw S. (1988). Human memory T lymphocytes express increased levels of three cell adhesion molecules (LFA-3, CD2, and LFA-1) and three other molecules (UCHL1, CDw29, and Pgp-1) and have enhanced INF-gamma production. J. Immunol. **140:** 1401–1407.

Seed B. (1987). An LFA-3 cDNA encodes a phospholipid linked membrane protein homologous to its receptor CD2. Nature **329:** 840–842.

Singer K.H., and Haynes B.F. (1987). Epithelial-thymocyte interactions in human thymus. Hum. Immunol. **20:** 127–144.

Spanopoulou E., Early A., Elliott J., Crispe N., Ladyman H., Ritter M., Watt S., Grosveld F., and Kioussis D. (1989). Complex lymphoid and epithelial thymic tumors in Thy 1-*myc* transgenic mice. Nature **342:** 185–189.

Springer T.A. (1990). Adhesion receptors of the immune system. Nature **346:** 425–434.

Springer T.A., Dustin M.L., Kishimoto T.K., and Marlin S.D. (1987). The lymphocyte function-associated LFA-1, CD2 and LFA-3 molecules: Cell adhesion receptors of the immune system. Ann. Rev. Immunol. **5:** 223–252.

Streuli M., Hall L.R., Saga Y., Schlossman S.F., and Saito H.

(1987). Differential usage of three exons generates at least five different mRNAs encoding human leucocyte common antigens. J. Exp. Med. **166:** 1548–1566.

Stutman O., Yunis E.J., and Good R.A. (1969). Thymus: An essential factor in lymphoid repopulation. Transplant. Proc. **1:** 614–615.

Towbin H., Staehelin T., and Gordon J. (1979). Electrophoretic transfer of proteins from polyacrylamide gels to nitrocellulose sheets: Procedure and some applications. Proc. Nat. Acad. Sci. USA **76:** 4350–4354.

Von Boehmer H. (1988). The developmental biology of T lymphocytes. Ann. Rev. Immunol. **6:** 309–326.

Williams A.F. (1988). The structure of Thy-1 antigen. *In:* Neuronal and glial proteins: Structure, function and clinical application (San Diego: Academic Press), pp. 49–69.

